# Impact of a peer-to-peer escape room activity in the learning of Human Physiology of medical students from the university of Málaga

**DOI:** 10.3389/fphys.2023.1242847

**Published:** 2023-08-30

**Authors:** D. Carrasco-Gomez, A. Chao-Écija, M. V. López-González, M. S. Dawid-Milner

**Affiliations:** ^1^ Department of Human Physiology, Human Histology, Pathological Anatomy and Physical Sport, University of Malaga, Malaga, Spain; ^2^ Autonomic Nervous System Unit, CIMES, School of Medicine, University of Málaga, Malaga, Spain

**Keywords:** medical physiology, peer-to-peer, escape room, gamification, physiology education, physiology teaching, medical student

## Abstract

Escape room’s popularity has raised over the past years among young adults. It creates a distended competitive environment, where participants collaborate to achieve a common objective through teamwork. We decided to apply this format as a teaching method for medical students at the University of Malaga, Spain. A peer-to-peer physiological cardiorespiratory escape room was designed by intern undergraduate students, collaborating within the Department of Human Physiology. This activity integrated the contents of the Human Physiology syllabus, which were organized into four stages that culminated in a final medical case. Intern students oversaw the design, promotion, preparation and execution of the activity, and were in charge of conducting the evaluation and follow up. The escape room was done in mid-December, after all theoretical and practical contents had been delivered, for four consecutive years, improving from each year’s experience. The target group for this activity were second year medical students, who were asked to team up freely in groups of four to six students before the start of the activity. The students in each group cooperated with each other while trying to solve the different puzzles and questions in each stage of the escape room. After the activity, the results of the final evaluation exam of these participants were compared against non-participants, who served as a control group. Qualitative feedback was also received from the participants *via* a special survey that was designed for this task. Results between 2020 and 2023 (three last activities) show that the final mark of the participants was significantly higher than in non-participants (6.39 ± 0.14 vs. 5.04 ± 0.2; *p* < 0.0007). The global exam mark also increased in the participants (5.43 ± 0.10 vs. 4.44 ± 0.15; *p* < 0.0007). A significant difference was observed in the performance in cardiovascular (*p* < 0.0007) and respiratory-related questions (*p* < 0.0007), which was substantial in the participants. The qualitative feedback received from the participants was mainly positive, indicating an overall acceptance of the format by the students. We conclude that escape room format with a peer-to-peer structure is an efficient teaching tool for medical students performed by medical students in the field of Human Physiology.

## 1 Introduction

The escape room gamification teaching format has become increasingly popular in recent years within the fields of medical education. This learning strategy has been tried in health sciences (Dugnol-Menéndez et al., 2021; Gerber and Fischetti, 2022; Gómez-Urquiza et al., 2022) as well as a learning strategy targeted to both medical residents (Schlegel and Radico, 2020; Khanna et al., 2021) and students (Liu et al., 2020; Podlog et al., 2020; Fusco et al., 2022; Hursman et al., 2022; Martin and Gibbs, 2022). The strategy to develop an escape room in a clinical setting usually involves creating a conductor and cohesive plot, designing problem-solving tasks that include clues to be used for future tasks, and employing a system to gather feedback from the participants, usually in the form of a survey (Dugnol-Menéndez et al., 2021; Gómez-Urquiza et al., 2022).

The design methodology of the escape room varies from work to work. While some authors prefer using physical objects and containers with combination locks (Liu et al., 2020), others prefer the use of computer software and augmented reality (Dugnol-Menéndez et al., 2021). This last strategy can be suitable for the development of more complex scripts that may serve to create a better immersive experience for the students.

This gamification method is usually positively received by students (Gómez-Urquiza et al., 2022), sometimes to the point that participants admittedly prefer them over more conventional learning methodologies (Liu et al., 2020; Dugnol-Menéndez et al., 2021). Qualitative feedback also seems to indicate that, in clinical settings, students perceive escape-room activities to highly prepare them for future potential theoretical or practical tasks, such as simulated clinical cases (García-Seoane et al., 2021). This is important in the context of the Spanish Medical Degree, as Medical Schools in Spain tend to use OSCE exams to evaluate clinical skills, which after the COVID-19 pandemic are also held online (García-Seoane et al., 2021).

We propose in this work a combination of this teaching tool with the concept of peer-to-peer learning. The objective of this methodology is that medical degree students review syllabus contents and receive feedback from their peers (Shenoy and Petersen, 2020). This learning strategy, in our work, can be better described as a near-peer tutoring learning system, as there is a difference in knowledge between the peers implicated in the activity (Shenoy and Petersen, 2020).

The School of Medicine of the University of Malaga offers in its curricular program three subjects dedicated to the study of physiology: General Physiology, which is centered on the study of physiology at a cellular level; Human Physiology I, which covers human cardiorespiratory, gastrointestinal and renal physiology; and Human Physiology II, which covers human neurophysiology and endocrinology. Our main objective for this study was to apply an escape-room teaching approach designed by students for students, on second year medical students of the University of Malaga, within the subject of Human Physiology I. Then, we validated the impact of this teaching tool by analyzing the academic performance of these second-year students, as well as their qualitative feedback.

## 2 Materials and methods

**FIGURE 1:**
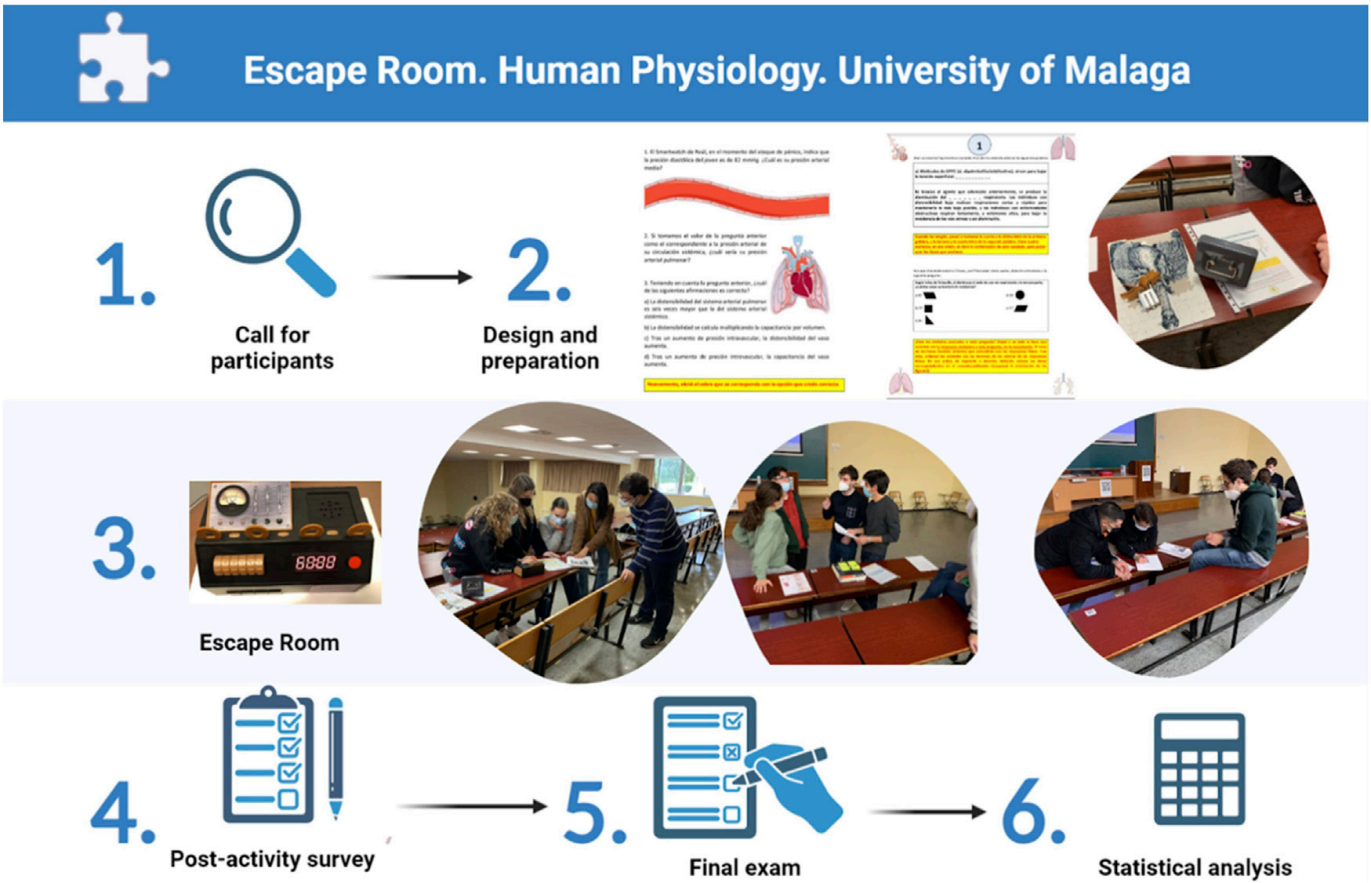
Workflow of the methodology of the peer-to-peer escape room project.

As represented in Figure 1, the escape room relied on 1) call for participants, released in late November, in which students were able to form groups of four to six people as they wished; 2) design and preparation of the activity, updated every year by the intern students’ committee; 3) execution of the escape room during the last week of December’s university schedule; 4) post-activity survey; 5) final exam, taken on the first week of February; 6) statistical analysis and conclusions An escape room activity was first conceived and designed by recruited intern students from the Human Physiology department of the University of Malaga. The activity was designed to test the contents of the syllabus related to Human Physiology I subject. These intern students already passed said subject with a high academic performance. Each intern student contributed to the design of the different stages of the activity, either by developing a suitable script or by providing different sets of materials to be used for the tasks.

**FIGURE 2:**
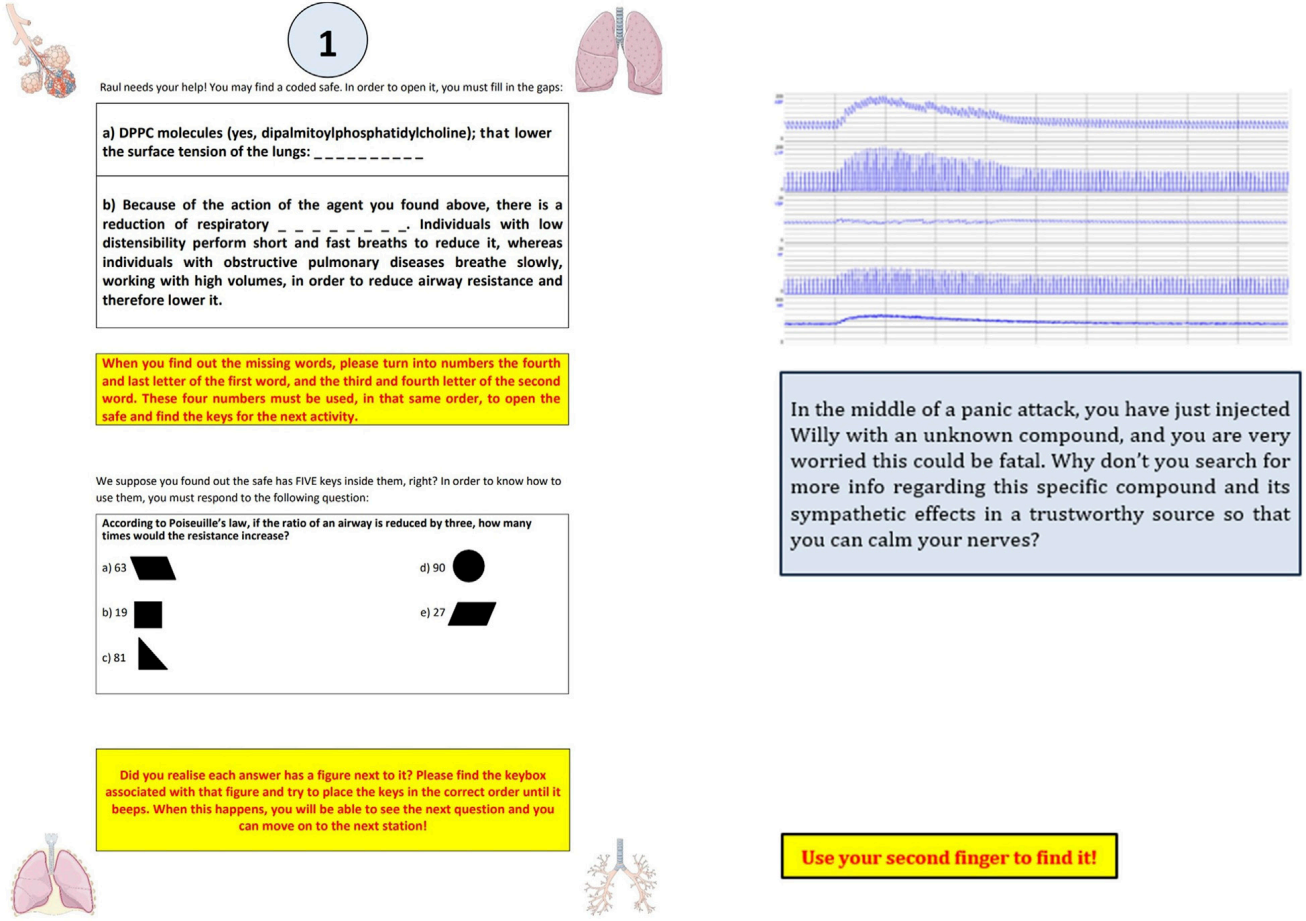
Some examples of specific tasks are provided. On the left, students must find out the word missing in order to get clues to open a box (topic: surfactant and its effect on work of breathing) or understand Poiseuille’s law and its implications in terms of airway resistance, flow and radius, linked to B2-agonists use [Respiratory stage]. On the right, students can find a simulation of continuous monitorization of blood pressure, heart rate, and other items, in order to identify which compound is administered, leading to other questions hidden in Guyton & Hall [Cardiovascular stage].

Five different stages were designed for this activity. The first four stages covered the contents of cardiovascular and respiratory physiology separately. The final stage was dedicated to an endgame task that the students had to complete in order to successfully solve the escape room activity. The plot of a fictional clinical case was devised to connect the various stages of the escape. This plot would be related to a patient with an unknown disease: the main goal of the students would be to gather enough clues from the first four stages so that they can obtain enough information to solve the final endgame task. This plotline was then divided into three different versions, which would be presented at different dates to the students. A summary of the tasks involved in each stage of the activity, as well as the different materials that were used, is presented in Table 1. Figure 2 depicts specific materials designed for the execution of the tasks.

**TABLE 1:** Description of the designed stages for the escape-room activity.

Stage	Topic	Tasks	Materials
I	Cardiovascular Part I	Explain sympathetic or vagal changes, explain in detail the biological mechanisms involved in those changes, work with arterial blood pressure and the specific properties of blood vessels	Paper sheets edited to show some of the clues and exercises, envelopes, combination lock boxes, key lock boxes, a jigsaw puzzle
II	Cardiovascular Part II	Explain sympathetic effects, work with fluid dynamics, work with and interpret ECG results, work with hydrostatic pressures	Paper sheets edited to show some of the clues and exercises, a modified Guyton book, combination lock boxes, paper sheet with ECG results
III	Respiratory Part I	Work with lung volumes, analyze tissue components of the blood-air barrier, work with plethysmography, analyze breathing patterns, describe and work with the concept of pulmonary dead space	Paper sheets edited to show some of the clues and exercises
IV	Respiratory Part II	Work with specific concepts related to respiration, explain changes in lung pressures, explain changes in blood flow, explain changes in airway resistance, work with lung volumes and the helium dilution technique	Paper sheets edited to show some of the clues and exercises, a jigsaw puzzle, several combination locks, interactive toy contraption from a game board with special keys to introduce several codes
V	Endgame	Final case. Students are required to solve a respiratory-physiology-related case. Students are required to use all the clues recovered in the previous stages. Students may know and apply the alveolar gas education. Students may be required to identify changes in the ventilation-perfusion ratio and apply the Shunt equation	Paper sheets edited to show some of the clues and exercises, a combination lock box containing several candy

The students were organized into different groups, which were given an introduction document with information about the fictitious storyline, a confidentiality consent document which they had to sign, and sheets of paper in case they would like to take any notes. Intern students from the department supervised the groups and the tasks of each stage, and were in charge of ensuring a correct workflow during the activity. These intern students in particular are characterized by having finished their second year of studies and by having passed the three Physiology subjects with high grades. They collaborate as undergraduate assistants and researchers in the department, either recruited for their high achievements or by voluntarily joining it.

After completing the escape room, the students had to anonymously answer a qualitative survey with questions related to their experience. This information was intended to serve as feedback for the organizers, and opened room to future improvements for the activity. Specific items of the survey are displayed in Table 2.

**TABLE 2:** Detailed description of the items evaluated through the qualitative survey. Items eight and nine presented in this table were repeated for each of the stages of the escape room activity.

Item	Type of answer
Gender	Qualitative dichotomous: male/female
Age	Quantitative variable. Positive integer
Previous qualifications	Qualitative nominal
Date of the activity	Qualitative nominal: Monday/Tuesday/Wednesday
Is it your first time in this type of activity? Have you ever done an escape room before?	Qualitative dichotomous: yes/no question
How high are your expectations regarding this activity?	Qualitative ordinal: Likert-type scale from one (very low) to five (very high)
And your previous knowledge of this subject before the start of this activity?	Qualitative ordinal: Likert-type scale from one (completely unprepared) to five (completely prepared)
How difficult did you find this stage overall?	Qualitative ordinal: Likert-type scale from one (very easy) to five (very difficult)
Did you consider yourself prepared for [stage]?	Qualitative dichotomous: yes/no question
Which stage did you feel more comfortable with?	Qualitative nominal: first and second stage (circulatory)/third, fourth and final stage (respiratory)/both/none
Which stage do you think you were less prepared to solve?	Qualitative nominal: first and second stage (circulatory)/third, fourth and final stage (respiratory)/both/none
Which stage do you consider to be more useful for your learning?	Qualitative nominal: first and second stage (circulatory)/third, fourth and final stage (respiratory)/both/none
Which stage, in your opinion, was less useful?	Qualitative nominal: first and second stage (circulatory)/third, fourth and final stage (respiratory)/both/none
Any comments about why a particular stage was more/less useful?	Open answer
In general, and after taking part in this activity, do you believe to be sufficiently prepared to solve this tasks?	Qualitative dichotomous: yes/no question
Do you believe taking part in this activity was somehow beneficial to you?	Qualitative dichotomous: yes/no question
What do you think should be improved?	Open answer
Describe your experience in five words or less	Open answer

At the end of the semester, the students were to pass a final exam to graduate from the subject. After the exam, marks obtained by the participants were compared against non-participants, which served as a control group. Each exam was evaluated and corrected by professors of the subject, with no relation with the developing process of the escape room activity nor with the intern students in charge of said development.

Results were gathered along three consecutive years, after a preliminary escape room experience took place in December 2019, in which all students were obliged to participate in the activity. While feedback received from this preliminary test was positive, the activity was perceived to last long by the last groups to enter the escape room, and some of these students were not interested in participating due to various diverse and personal reasons. Therefore, as the authors did not want to conceive it as an exam or a mandatory activity to pass the subject, we designed the final activity as voluntary, and inserted it in the subjects’ curricula, thus allowing us to also have a control group to study its effect.

Between 2020 and 2023, three escape rooms were performed, having 245 participants compared to 151 non-participants. In total, 396 students took the exam in February of the corresponding year. We intended to evaluate the escape room’s impact on the performance of students that attended the activity voluntarily, opposed to the result of those students that did not attend. In addition, subjective feedback about preparedness, subjective perception of the level of the activity and overall opinion was collected. This would serve as real life data supporting or denying the efficacy of gamification in escape room format to teach Human Physiology for second year medical students in the University of Malaga.

This study in no case can be considered as clinical experimentation in human beings. The ethical principles contained in the Declaration of Helsinki of the World Medical Association (2013) have been followed at all times. The escape participants granted a valid written informed consent. Personal data are confidential and treated in accordance with the provisions of Regulation (EU) 2016/679 “General Data Protection” and Organic Law 3/2018, of December 5, on “Personal Data Protection” The study was reviewed and approved by Comité Ético de Experimentación de la Universidad de Málaga (CEUMA).

### 2.1 Statistical analysis

Data processing was carried out using statistical package GraphPad Prism 9. Descriptive analysis of continuous quantitative variables was performed, obtaining mean, standard deviation, minimum and maximum for each parameter. For analytical statistics, data were grouped into two comparison groups according to the participation or not in the escape room. The significance level was established at *p* < 0.05. The Shapiro test for normality rejection was first used to test normality in each of the analyzed distributions. For each pair of distributions, if normality could not be rejected for both of the distributions, a parametric unpaired *t*-test was used to compare them. Otherwise, a non-parametric Mann–Whitney *U* test was used instead. Bonferroni correction for seven independent comparisons was applied to control the false positive rate of the data. In all cases, graphical representations in the form of box plot have been used.

## 3 Results

### 3.1 Qualitative results from survey

**FIGURE 3:**
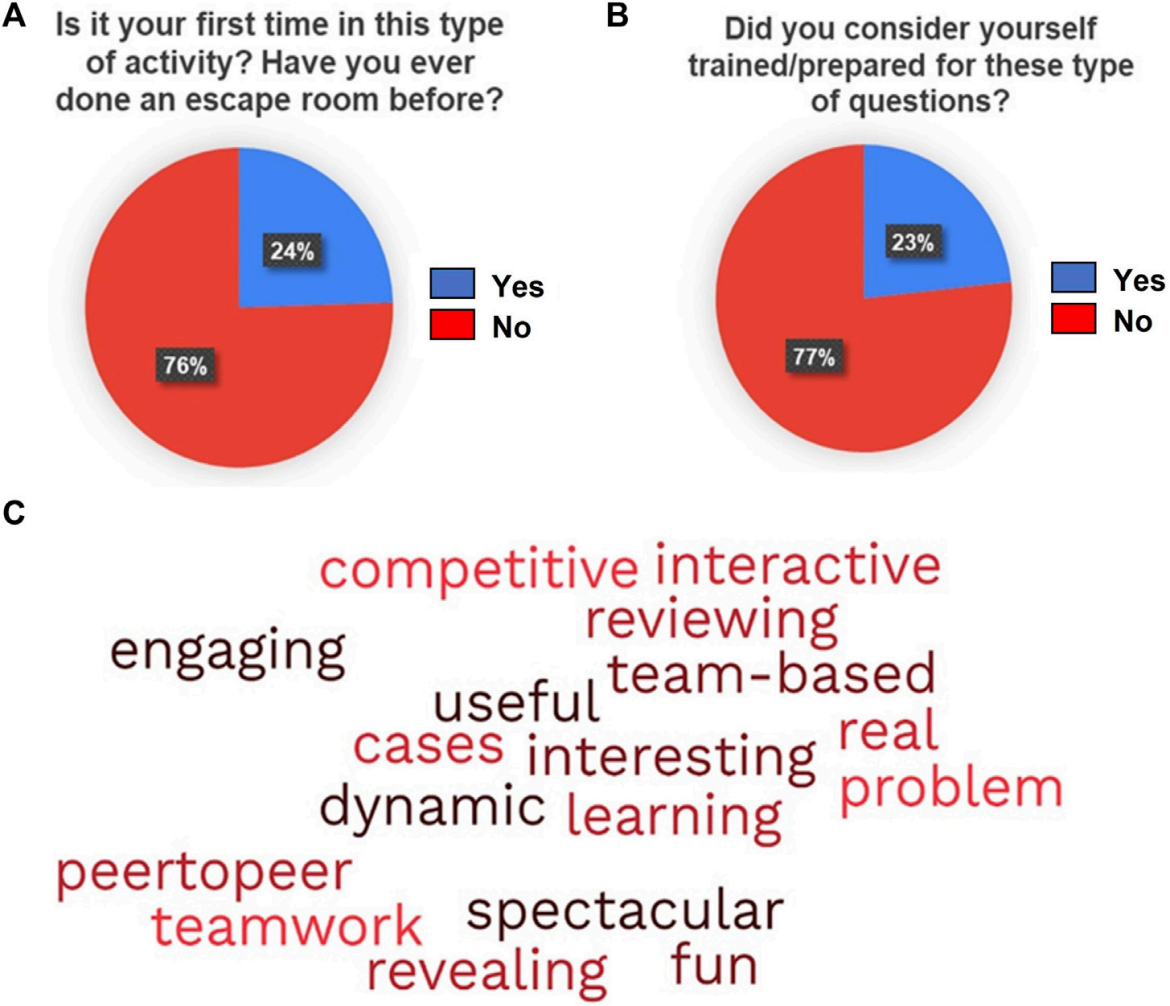
Answers obtained from the qualitative survey from **(A)** item V (shown as percentage), **(B)** item XV (shown as percentage) and **(C)** item XVIII (shown as word cloud).

**FIGURE 4:**
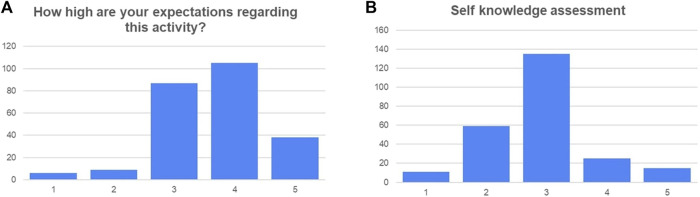
Response distribution regarding **(A)** previous expectations and **(B)** self-knowledge before the start of the activity.

Results from qualitative assessment of the activity with the discussed survey are summarized in the following figures. The percentage distribution for participants’ gender was 77% for female and 23% for male. Figure 3A summarizes the distribution for item V, with 75% of the students being new to this kind of activity. The answer distribution regarding previous expectations and self-knowledge assessment are depicted in Figure 4.

**FIGURE 5:**
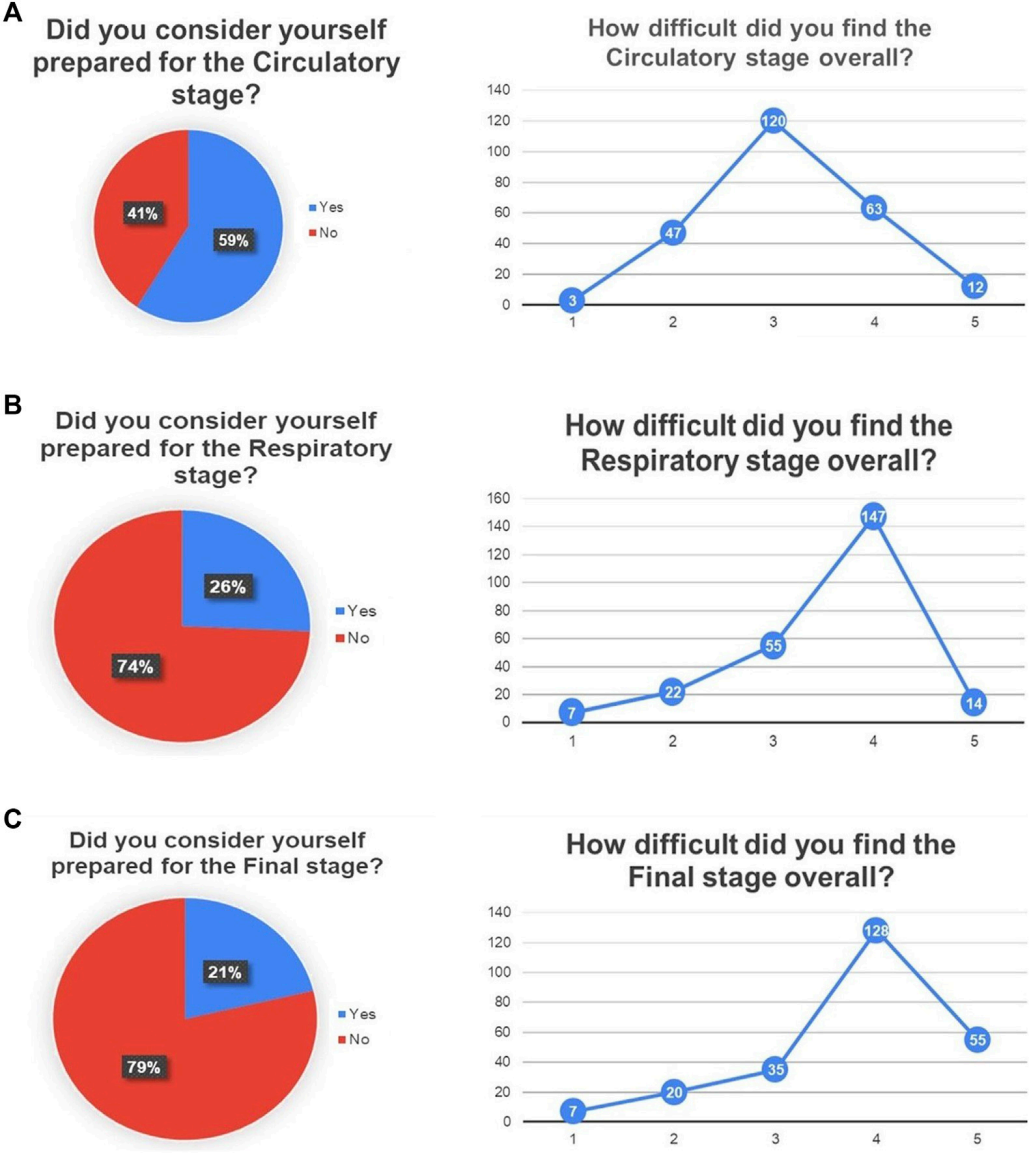
Qualitative evaluation for each stage of the escape room activity, regarding difficulty and self-awareness of previous preparation: **(A)** Cardiovascular, **(B)** Respiratory and **(C)** Final stage.

**FIGURE 6:**
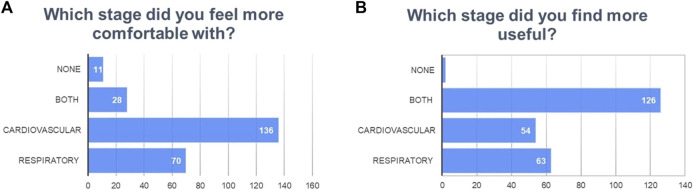
Answer distribution for overall qualitative evaluation of the stages of the escape room.

Figure 5 depicts the difficulty evaluation and self-awareness assessments given by each student to each of the stages of the escape room activity. The modes for each distribution regarding the first of these two items are three (cardiovascular stage) and four (respiratory stage and final stage). While 59% of the students considered themselves prepared to solve the cardiovascular stage, 26% kept these thoughts regarding the respiratory stage, and 21% did for the final stage. The majority of students felt more comfortable with the cardiovascular stage, but considered both stages equally useful, as summarized in Figure 6.

**FIGURE 7:**
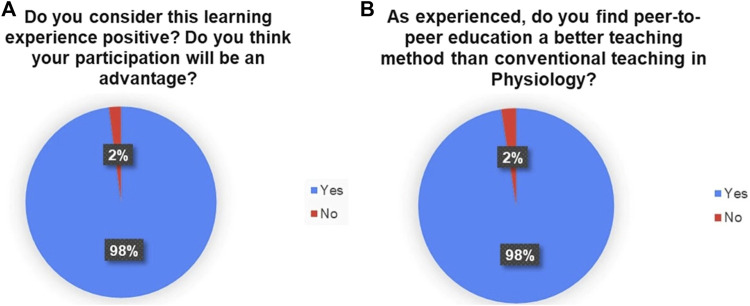
Percentage distribution of answers regarding the benefits of the escape room and the peer-to-peer teaching method.

At the end of the activity, more than 70% of the students did not consider themselves to have been sufficiently prepared for these tasks. Regarding the usefulness of the activity, 98% of the students found the activity positive and advantageous for their learning. The majority of the students preferred peer-to-peer teaching over traditional methods. These results are summarized in Figure 3B and Figure 7. Figure 3C depicts a word cloud created with the open answers to the last item of the survey.

### 3.2 Participants performance in the final exam

**FIGURE 8:**
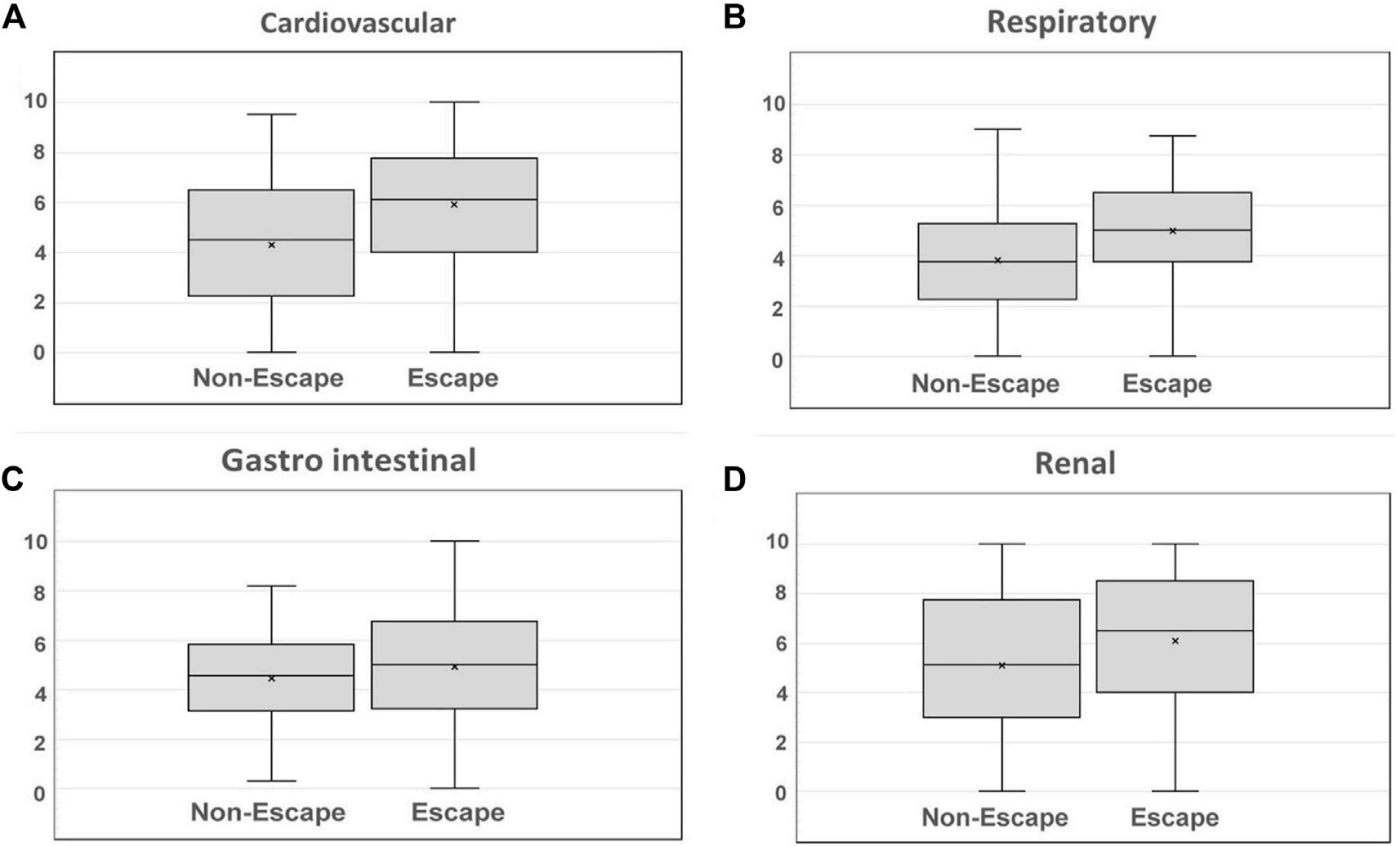
Academic performance shown for individual areas of knowledge: **(A)** Cardiovascular, **(B)** Respiratory, **(C)** Gastro intestinal, and **(D)** Renal physiology.

Mean academic performance from both participants and non-participants is summarized in Table 3. Significant differences were obtained for three of the four main analyzed areas of knowledge, with a higher performance corresponding to the participants group. These differences are also summarized in Figure 8.

**TABLE 3:** Means and Standard Errors of the Mean (SEM) of marks obtained in the final exam for every evaluated item, as well as the final mark. Significances are given with a confidence level of 0.95, and result from applying the parametric *t*-test when normality could not be rejected, or the Mann-Whitney U test otherwise. Both their raw and adjusted versions (Bonferroni correction for seven independent comparisons) are shown.

Item	Participants (n = 245) (Mean ± SEM)	Non-participants (n = 151) (Mean ± SEM)	*p*-value	Adjusted *p*-value
Cardiovascular	5.93 ± 0.15	4.30 ± 0.21	*p* < 0.0001	*p* < 0.0007
Respiratory	5.01 ± 0.13	3.81 ± 0.18	*p* < 0.0001	*p* < 0.0007
Gastrointestinal	4.92 ± 0.16	4.21 ± 0.22	*p* = 0.0155 (*p* < 0.05)	*p* = 0.1085
Renal	6.06 ± 0.18	5.08 ± 0.24	*p* = 0.0015 (*p* < 0.01)	*p* = 0.0105 (*p* < 0.05)
Test questions	5.38 ± 0.10	4.52 ± 0.15	*p* < 0.0001	*p* < 0.0007
Global	5.43 ± 0.10	4.44 ± 0.15	*p* < 0.0001	*p* < 0.0007
Final mark	6.39 ± 0.14	5.04 ± 0.20	*p* < 0.0001	*p* < 0.0007

**FIGURE 9:**
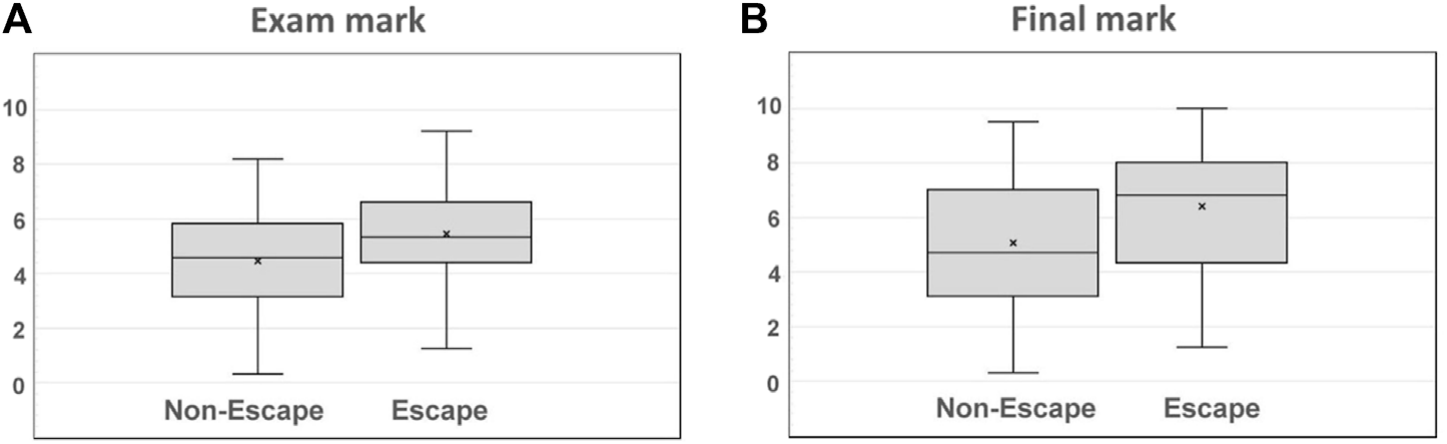
Overall academic performance for the **(A)** exam mark and **(B)** final mark.

Figure 9 shows differences observed for the overall mark of the exam as well as the final mark for the entire subject. Significant differences were observed once again between both groups, with the highest performance belonging to the participants group.

## 4 Discussion

Results obtained from the group comparison reveal an overall better performance of participants when compared against non-participants. This increase was especially evident in the cardiovascular physiology area, in which participants showed a significantly higher academic performance when compared with non-participants. A higher performance was also observed in areas related with respiratory physiology.

Even though significant differences were found for all the items, no significant differences were observed between both groups in areas related with gastrointestinal physiology after applying a Bonferroni correction to the results. We attribute this phenomenon to the fact that the contents of the escape-room activity were not designed to cover contents within this area. However, a lesser significant difference between both groups was observed when the academic performance within renal physiology questions was studied, with a higher performance attributed to the participants of the escape-room activity. We theorize that this increase in performance was due to the fact that some questions with partial relation to renal physiology were included in the problem-solving tasks of the circulatory physiology stages of the escape room.

Overall, the global mark of the exam, as well as the final mark of the whole subject, was significantly higher in participants. This served as an indicator of the global academic performance of these students, showing that the escape room activity had some influence not only in specific areas of the exam, but also on the overall results.

Qualitative results obtained from the survey, overall show a wide acceptance of the activity. The immense majority of the students seemed to agree regarding the overall usefulness of the peer-to-peer escape room. The activity also served as a self-awareness diagnostic tool, as the students were able to identify if they had a lack of previous preparation for the activities.

Apart from the experience obtained, it served as an informal way of assessing each students’ knowledge in a non-evaluating setting, therefore each participant could draw conclusions on their knowledge up to that date and adapt their efforts and focus on the topics of circulatory and respiratory physiology covered along the escape room. This was done more than 1 month ahead of the final exam, thus leaving time for the student to apply the experience to their study program.

Additionally, we would like to raise awareness of several limitations our study may have. Indeed, it is reasonable to hypothesize that making the escape room activity voluntary generated a selection bias on the sample. As true as it is, the first escape room that was performed included everyone and therefore could not allow us to measure its influence on students. On the other hand, randomly selecting the students that can participate would be statistically and scientifically understandable, although flagrantly unfair for students enrolled in the subject. As a result, the question that arises from this limitation is what percentage of the difference between groups (participants vs non-participants) could be explained by a “good student” bias? As this remains uncertain, we understand the implementation of the escape room as positive because of the feedback provided by participants, which is undoubtedly in favor of it.

During the design and execution of the escape room format, some problems were encountered along the way, such as intern student recruitment, date selection, request for permission to use the facilities, coordination with student representatives in order to ease communication prior to the escape room to set up the groups (designed by the students, so it would be a more desired environment for them) and schedule, and of course, COVID-pandemic, which was unexpected and forced the authors to introduce additional measures to the activity such as social distancing protocols and the use of sanitizers.

Another limitation that can be identified is a high percentage of students that did not take the exam in February (27% of enrolled students). As a result, the impact of the escape room lacks data from all these students, which constitutes a high percentage of those that did not participate in the escape room, thus hypothetically underestimating the impact of the activity. Lastly, any exam implies intrinsic variability among each student, as it is taken only once, therefore includes many other variabilities apart from knowledge, such as mental health status, time management or stress, among others.

## 5 Conclusion

The peer-to-peer escape room teaching approach was well received by the participants and significantly improved their academic performance. We propose to extend this methodology to other preclinical subjects, as well as to conduct further research when applied to clinical subjects.

## Data Availability

The raw data supporting the conclusion of this article will be made available by the authors, without undue reservation.
